# Multidrug resistant Enterobacteriaceae and extended spectrum β-lactamase producing *Escherichia coli*: a cross-sectional study in National Kidney Center, Nepal

**DOI:** 10.1186/s13756-015-0085-0

**Published:** 2015-10-26

**Authors:** Kamlesh Kumar Yadav, Nabaraj Adhikari, Rama Khadka, Anil Dev Pant, Bibha Shah

**Affiliations:** Department of Microbiology, Kantipur College of Medical Science, Sitapaila, Kathmandu, Nepal; Consultant pathologist, National Kidney Center, Vanasthali, Kathmandu, Nepal; Quality Control Department, Qmed Formulations Pvt. Ltd., Chhaling, Bhaktapur, Nepal

**Keywords:** Antibiotic resistance, *E. coli*, Enterobacteriaceae, ESBL, Multidrug resistance, Nepal, Urine, UTI

## Abstract

**Background:**

Emergence of antibacterial resistance and production of Extended spectrum β-lactamases (ESBLs) are responsible for the frequently observed empirical therapy failures. Most countries have experienced rapid dissemination of ESBLs producing Enterobacteriaceae isolates, particularly *E. coli* and *Klebsiella pneumoniae*. ESBLs are clinically significant and when detected, indicate the need for the use of appropriate antibacterial agents. But antibacterial choice is often complicated by multi-resistance.

**Methods:**

This study was carried from June to November 2014 to study the multidrug resistant (MDR) Enterobacteriaceae and ESBL producing *E. coli* among urine isolates in hospital setting. Isolates from urine samples were primarily screened for possible ESBL production followed by phenotypic confirmation. Antibiotic susceptibility testing (AST) was done by Kirby Bauer disk diffusion method following Clinical and Laboratory Standard Institute (CLSI) guidelines.

**Results:**

Out of 450 urine samples processed, 141 significant growths were obtained including 95 Enterobacteriaceae isolates with 67 *E. coli*. Among Enterobacteriaceae, 92 (96.84 %) were recorded as MDR and 18 (26.87 %) *E. coli* were confirmed as ESBLs producers.

**Conclusions:**

Using the phenotypic confirmatory test forwarded by the CLSI, relatively significant *E. coli* isolates tested were ESBL producers. Also high numbers of MDR organisms were isolated among Enterobacteriaceae. Isolates showed significant resistance to the commonly prescribed drugs. These findings suggest for further study in this field including the consequences of colonization with MDR and ESBL-producing bacteria both in the community and in the hospital setting.

## Background

Urinary tract infection (UTI) is a common disease ailment among Nepalese population as well as one of the commonest nosocomial infection [1]. Because of the evolving and continuing antibiotic resistance phenomenon, regular monitoring of resistance patterns is necessary to improve guidelines for empirical antibiotic therapy [2]. Uropathogens have developed resistance to commonly prescribed antimicrobial agents; this severely limits the treatment options of an effective therapy. One of the important resistance mechanisms is production of enzymes destroying the drug β-lactam antibiotics. To date several types of β-lactamases have been characterized depending on the characteristic and hydrolytic activity. Extended spectrum β-lactamases (ESBLs) is one of the important groups of β-lactamases [3].

ESBLs are the enzymes that have the ability to hydrolyze and cause resistance to various types of newer β-lactam antibiotics, including the expanded-spectrum (or third generation) cephalosporins (eg. cefotaxime, ceftriaxone, ceftazidime) and monobactams (eg. aztreonam), but not the cephamycins (eg. cefoxitin and cefotetan) and carbapenems (eg. imipenem, meropenem and etrapenem) [4]. These enzymes are sensitive to β-lactamase inhibitors (sulbactam, clavulanic acid, and tazobactam) [5].

A large number of outbreaks of the infections which are caused by ESBL producing organisms have been described in every continent of the globe [5]. There is ample evidence to suggest the spread of ESBL infections is higher in resource poor countries [6]. Major risk factors for colonization or infection with ESBL producing organisms are long term antibiotic exposure, prolonged intensive care unit (ICU) stay, nursing home residency, severe illness, residence in an institution with high rates of ceftazidime and other third generation cephalosporin use and instrumentation or catheterisation [7].

*E. coli*, that can produce ESBLs, has arisen and disseminated worldwide as an important cause of both nosocomial and community infections and nowadays represents a major threat. Early identification of potential ESBL carriers is the first step to withhold the dispersal of these microorganisms and to avoid possible complications [8]. Since, ESBL production is usually plasmid mediated, it is possible for one specimen to contain both ESBL producing and non ESBL producing cells of the same species. This suggests that for optimal detection, several colonies must be tested from a primary culture plate [7]. Adequate detection of ESBL-producing strains is crucial for appropriate choice of antimicrobial therapy and infection control measures [9].

MDR Enterobacteriaceae has been frequently reported from different parts of the world as an emergence of treatment problem. Antibiotics given empirically without proper antibiotic susceptibility testing are one of the major causes for the development of MDR. So, to ensure appropriate therapy, current knowledge of the organism that causes UTI and their antibiotic susceptibility is mandatory [10]. The dissemination of ESBL-producing Enterobacteriaceae in the hospital setting is a problem with major therapeutic and epidemiological consequences [11]. This study was aimed to investigate the current situation of ESBL-producing *Escherichia coli* among Enterobacteriaceae isolates and sensitivity pattern of isolates toward various chemotherapeutic agents.

## Methods

### Sample

This is a cross-sectional study conducted from June to November 2014 in National Kidney Center, Vanasthali, Kathmandu, Nepal. The study population included patients visiting the hospital suspected of UTIs and patients undergoing dialysis in the hospital.

Patients included in the study were given pre-labelled (date, time, identification code, age and sex), leak proof, sterile, screw-capped container to collect the mid-stream urine (MSU) sample. Urine samples from all age group were included in the study. Samples those held for more than two hours at room temperature and those without proper labelling were excluded from the study.

### Laboratory assessment

The collected urine specimens were processed in the Microbiology laboratory within 2 h of collection. Urine samples were streaked directly on MacConkey agar (MA) and Blood agar (BA) plates. These plates were incubated at 37 °C aerobically and after overnight incubation, they were checked for bacterial growth. The Gram negative isolates were identified by their colony morphology, Gram staining characteristics, catalase test, oxidase test, and other relevant biochemical tests as per standard laboratory methods of identification.

Antibiotic susceptibility testing of bacterial isolates was done by Kirby Bauer disk diffusion method following CLSI guidelines using Mueller Hilton Agar (MHA) [12]. The discs were taken from HiMedia Laboratories (India). The followings are the concentrations of drugs used for disc diffusion testing: amikacin (30 *μ*g), cefalexin (30 *μ*g), cefixime (5 *μ*g), cefotaxime (30 *μ*g), ceftazidime (30 *μ*g), ceftriaxone (30 *μ*g), ciprofloxacin (5 *μ*g), cotrimoxazole (23.75 *μ*g sulfamethoxazole/1.25 *μ*g trimethoprim), doxycycline (30 *μ*g), imipenem (10 *μ*g), nalidixic acid (30 *μ*g), nitrofurantoin (300 *μ*g), Norfloxacin (10 *μ*g), and ofloxacin (5 *μ*g). An isolate was considered as MDR if it was resistant to three or more drugs of different classes/groups of antibiotics.

### ESBL detection

All the *E. coli* isolates were subjected to the screening test for ESBL detection. Screening test for ESBL detection was done according to the CLSI guidelines [12]. Isolates showing inhibition zone size of ≤ 22 mm with ceftazidime (30 μg), ≤ 25 mm with ceftriaxone (30 μg), and ≤ 27 mm with cefotaxime (30 μg) were interpreted as screening test positive for ESBL production.

For the confirmatory test for ESBL, two or three colonies of organisms were suspended in 0.5 ml of sterile broth and the turbidity matched to 0.5 McFarland. Using a sterile cotton swab the broth culture was uniformly swabbed on MHA. All the *E. coli* isolates which were resistant to at least ceftazidime, ceftriaxone and/or cefotaxime were subjected to the ESBL confirmatory test using ceftazidime (30 μg) and ceftazidime-clavulanic acid (30 μg + 10 μg) and the cefotaxime (30 μg) and cefotaxime-clavulanic acid (30 μg + 10 μg) combination disks. The tests were interpreted according to CLSI guidelines and a difference of 5 mm between zone of inhibition of a single disk and in combination with clavulanic acid (inhibitor) was confirmed to be produced by an ESBL positive isolate.

## Results

Out of 450 urine samples processed in the laboratory, growth on MA and/or BA was obtained in 141 (31.33 %) urine samples (Table 1). Highest number of isolates was from the sample of patients with age above 60 years.

**Table 1:** Age and gender wise distribution of isolates

Age group	Male		Female		Total	
	Sample	Growth (%)	Sample	Growth (%)	Sample	Growth (%)
≤ 20	13	1 (1.79)	13	8 (9.41)	26	9 (6.38)
21 – 40	45	16 (28.57)	89	24 (28.24)	134	40 (28.37)
41 – 60	79	18 (32.14)	81	22 (25.88)	160	40 (28.37)
≥ 61	68	21 (37.50)	62	31 (36.47)	130	52 (36.88)
Total	205	56 (100)	245	85 (100)	450	141 (100)

Among the isolates (*n* = 141) 41 (29.08 %) were Gram positive organism and 100 (70.92 %) were Gram negative organisms (Table 2). *S. aureus* and *E. coli* were the most predominant organism among Gram positive and Gram negative respectively. Out of 100 Gram negative organisms, 95 (95 %) were of Enterobacteriaceae family. *E. coli* was the most predominant genera of Enterobacteriaceae followed by *Klebsiella* spp, *Proteus* spp and *Citrobacter* spp. *E. coli* was isolated in 67 (47.52 %) samples. Other Gram negative organisms isolated were *Pseudomonas* spp and *Neisseria* spp.

**Table 2:** Microbiological profile of urinary isolates

	Isolates	Number (%)
Gram positive	Coagulase-negative staphylococci	18 (12.77)
	*S. aureus*	15 (10.64)
	*Enterococcus* spp	8 (5.67)
Sub total		41 (29.08)
Gram negative	*E. coli*	67 (47.52)
	*Klebsiella* spp	24 (17.02)
	*Proteus* spp	3 (2.13)
	*Citrobacter* spp	1 (0.71)
	*Pseudomonas* spp	2 (1.42)
	*Neisseria* spp	3 (2.13)
Sub total		100 (70.92)
Total		141 (100)

The resistance of Enterobacteriaceae isolates against a spectrum of 14 selected antimicrobial agents of different classes were analyzed (Table 3). Enterobacteriaceae isolates showed variable result in their antibiotic sensitivity pattern against commercial antibiotic discs tested. According to the susceptibility pattern imipenem (92.63 %) was the most effective antibiotics against Enterobacteriaceae followed by the amikacin (82.11 %) and nitrofurantoin (57.89 %). Out of 14 antibiotics tested, 11 were found effective only for less than half of the Enterobacteriaceae isolates. Among the 95 Enterobacteriaceae isolates none of them were sensitive to all antibiotics tested.

**Table 3:** Antimicrobial resistance amongst Enterobacteriaceae (*n* = 95)

Antibiotics class	Antibiotics	Resistance no. (%)
Aminoglycoside	Amikacin	17 (17.89)
Beta-lactams	Imipenem	7 (7.37)
	Cefalexin	79 (83.16)
	Cefixime	72 (75.79)
	Cefotaxime	71 (74.74)
	Ceftazidime	79 (83.16)
	Ceftriaxone	65 (68.42)
Nitrofuran	Nitrofurantoin	40 (42.11)
Quinolones/ Fluoroquinolones	Ciprofloxacin	58 (61.05)
	Nalidixic Acid	77 (81.05)
	Norfloxacin	61 (64.21)
	Ofloxacin	59 (62.11)
Sulfonamide	Co-Trimoxazole	59 (62.11)
Tetracycline	Doxycycline	56 (58.95)

Seventy three varied patterns of the antibiotic susceptibility were observed among the enterobacteriaceae isolates against the 14 different antibiotics. Each of these patterns was common in one or up to eight isolates which were analyzed. Enterobacteriaceae isolates include 67 *E. coli*, 24 *Klebsiella* spp, 3 *Proteus* spp. and one *Citrobacter* spp. Out of 95 Enterobacteriaceae, 92 (96.84 %) isolates were MDR (Table 4). Sixty four (95.52 %) isolates of *E. coli* and all isolates of *Klebsiella* spp, *Proteus* spp and *Citrobacter* spp were detected as MDR.

**Table 4:** MDR trend in Enterobacteriaceae family

Organisms	Total number	MDR strains (%)
*E. coli*	67	64 (95.52)
*Klebsiella* spp	24	24 (100.00)
*Proteus* spp	3	3 (100.00)
*Citrobacter* spp	1	1 (100.00)
Total	95	92 (96.84)

**Fig. 1:**
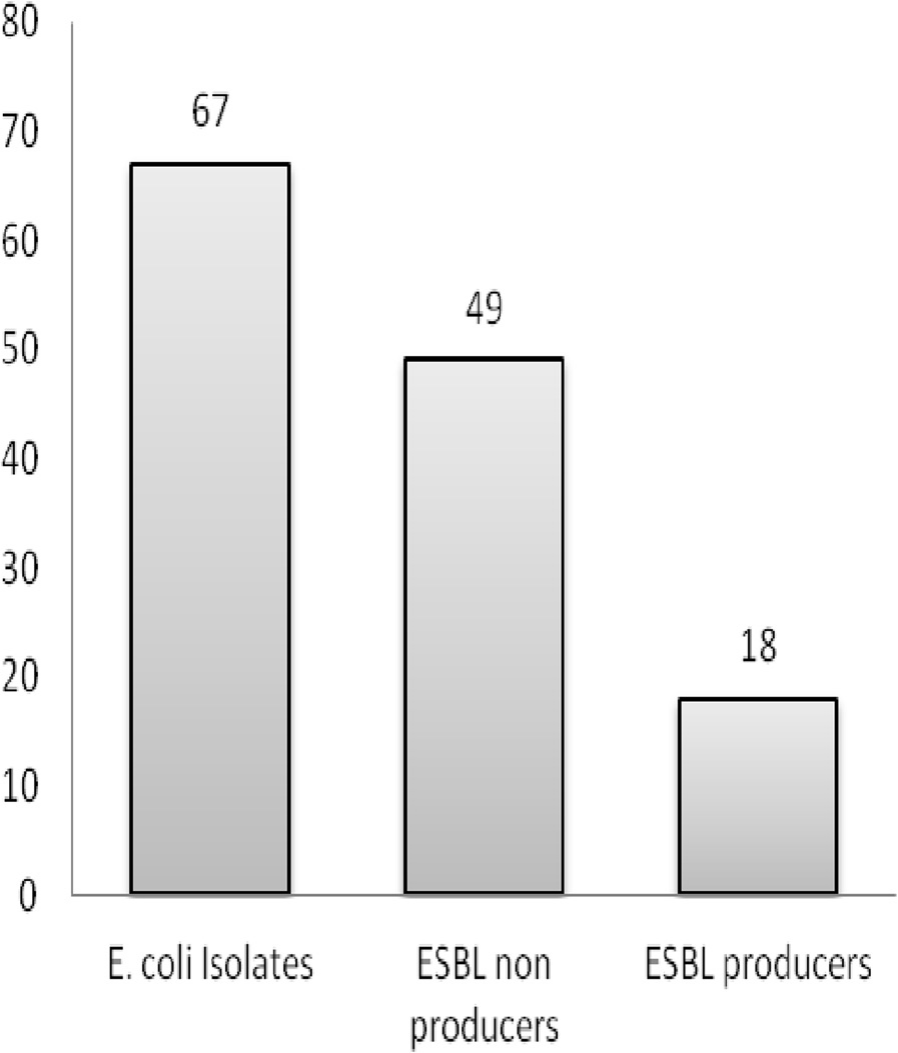
ESBL production profile of *E. coli* isolates

Out of total 67 strains of *E. coli*, which were screened for ESBL production, 53 (79.10) isolates were positive. All the *E. coli* isolates which were resistant to at least ceftazidime, ceftriaxone and/or cefotaxime were considered as screening positive isolates. After performing phenotypic confirmation test 18 (26.87 %) *E. coli* isolates were confirmed as ESBL producers (Fig. 1).

ESBL positive *E. coli* showed high degree of resistance to the antibiotics tested. All 18 ESBL positive *E. coli* isolates were resistant (100 %) to cefotaxime, ceftazidime and ceftriaxone (Table 5). These isolates also showed high resistance to other antibiotics as well. More than 60 % of ESBL positive isolates were resistant to 11 antibiotics out 14 antibiotics used for test. Most effective drug for the ESBL positive isolates was amikacin, to which all (100 %) isolates were susceptible. Amikacin was followed by imipenem (94.44 %) and nitrofurantoin (72.22 %).

**Table 5:** Antimicrobial resistance among ESBL producing *E. coli* (*n* = 18)

Antibiotics	Resistance no. (%)
Amikacin	0 (0.00)
Imipenem	1 (5.56)
Cefalexin	17 (94.44)
Cefixime	17 (94.44)
Cefotaxime	18 (100.00)
Ceftazidime	18 (100.00)
Ceftriaxone	18 (100.00)
Ciprofloxacin	16 (88.89)
Co-Trimoxazole	11 (61.11)
Doxycycline	13 (72.22)
Nalidixic Acid	17 (94.44)
Nitrofurantoin	5 (27.78)
Norfloxacin	17 (94.44)
Ofloxacin	16 (88.89)

**Fig. 2:**
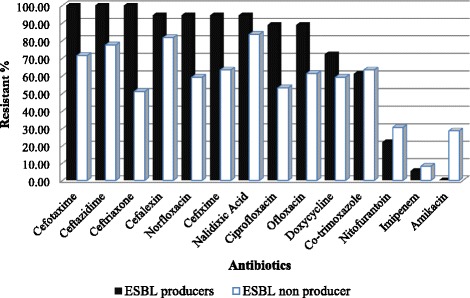
Comparison of ESBL producers and non producers to the antibiotics tested

High degree of resistance was shown by ESBL producers than ESBL non producers (Fig. 2). Only in case of cotrimoxazole, nitrofurantoin, imipenem and amikacin ESBL non producers showed a bit higher resistance than ESBL producers. Most effective drug for the ESBL non producers was found to be imipenem which was susceptible to 45 (91.84 %) out of 49 isolates, followed by amikacin susceptible to 35 (71.43 %) isolates and nitrofurantoin susceptible to 34 (69.39 %) isolates.

## Discussion

This study was aimed to investigate ESBL-producing *E. coli* among Enterobacteriaceae isolates and sensitivity pattern of isolates toward various chemotherapeutic agents. Organisms producing ESBLs are clinically relevant and remain an important cause of failure of therapy with cephalosporins. ESBLs are primarily produced by the Enterobacteriaceae family, in particular *K. pneumoniae* and *E. coli*. Bacteria harbouring ESBLs may also acquire and most often exhibit additional resistances to other antimicrobial classes such as the quinolones, tetracyclines, cotrimoxazole, trimethoprim, and aminoglycosides, which further limits therapeutic options and thus pose a therapeutic dilemma [13].

Significant growth was obtained in 31.33 % urine culture samples. The majority of urine specimens showed no growth (68.67 %). The possible cause of low rate of growth positivity might be due to urine samples obtained from patients on antibiotics therapy, infection due to slow growing organisms or due to those organisms that were not able to grow on the routine media used [1, 14]. Number of female patients requesting for urine culture was higher than the male patients. Significant microbial growth was higher in case of female than in male. Urethral opening in females, short urethra and complicated physiology especially during pregnancy can be considered as reason [15]. Female patients requesting for urine culture was higher, than the male patients, in age group of 21–40 years this may be because this age group consists sexually active women. Frequent or recent sexual activity is the most important risk factor for UTIs in young women. Nearly 80 % of all UTIs in premenopausal women occur within 24 h of intercourse. UTIs are very rare in celibate women. Certain types of contraceptives can also increase the risk of UTIs [16].

Numbers of gram negative organisms isolated were much higher than the gram positive. Similar predominance of gram negative organism in urine sample has been observed by other researchers too [17, 18]. *Staphylococcus aureus,* Coagulase-negative staphylococci (CoNS) and *Enterococcus* spp. were the gram positive organisms isolated. *E. coli*, *Klebsiella* spp., *Proteus* spp., *Citrobacter spp*., *Pseudomonas* spp. and *Neisseria* spp. were the gram negative organisms isolated. Among the gram negative organisms, Enterobacteriaceae were most frequent, 95 out of 100 gram negative organisms. Members of Enterobacteriaceae are more likely to cause UTIs than other organisms. In various studies predominant organisms isolated in UTI cases is Enterobacteriaceae [17, 19, 20].

Antibiotic susceptibility pattern shown by the Enterobacteriaceae isolates were variable. Imipenem was the most effective antibiotic as 92.63 % of isolates were susceptible, followed by amikacin (82.11 %). Isolates were comparatively less susceptible to cephalosporins than other antibiotics. Resistance to β-lactams in Enterobacteriaceae is mainly due to the production of β-lactamases, which may be encoded either chromosomally or on plasmids [4]. Out of 14 antibiotics used, 11 were found effective to only less than half of the isolates. Majority (96.84 %) of Enterobacteriaceae isolates were found to be MDR. Thakur et al. [21] has observed 64.04 % MDR Enterobacteriaceae and 73.68 % MDR *E. coli* isolates. The widespread use of antibiotics could be associated with the selection of antibiotic resistance mechanisms in pathogenic and non pathogenic isolates of *E. coli* [22]. MDR isolates were more in females than in males and common in age group ≥ 61 years.

Over the past few years, the prevalence of ESBL producing strains among clinical isolates varies greatly with different geographic regions and rapidly changing over time [23]. In this study, phenotypically 18 (26.87 %) isolates were confirmed as ESBL producers *E. coli* isolates. ESBL positive *E. coli* was distributed equally among male and female. Highest number of ESBL producers *E. coli* was obtained from the patients of age above 60 years. Other studies have also shown that ESBL isolates are encountered more frequently in the elderly, according to Roshan et al. [24], Shah et al. [25] and Rajan and Prabavathy [26] majority of isolates were from patients between 40 to 70 years, 50 to 60 years and 51 to 70 years respectively. The ESBLs-producing *E. coli* were most frequent in older age group in this study; it can be due to the reason that older patients are immunocompromised and more prone to infections by resistant organisms [27]. Nosocomial infections caused by ESBL producing pathogens are associated with risk factors such as elderly age, prolonged hospitalization, previous antibiotic use, and presence of invasive devices [28].

All ESBL positive *E. coli* strains were resistant to cefotaxime, ceftazidime and ceftriaxone. This outcome is in agreement with the study done by Islam et al. [29]. Similarly all *E. coli* isolates were resistant to cefotaxime and ceftriaxone in a study by Sompolinsky et al. [30] and to ceftazidime and ceftriaxone in a study by Chander and Shrestha [6]. High percentage of resistance to cefotaxime (99.2 %), ceftazidime (99.2 %) and ceftriaxone (99.5 %) was observed by Wani et al. [31]. ESBL positive isolates also showed high degree of resistance to other antibiotics like cefalexin, norfloxacin, cefixime, nalidixic acid, ciprofloxacin and ofloxacin. Aminoglycosides have good activity against clinically important gram negative bacilli [32]. Aminoglycosides are very important group of antibiotics with activity against many gram-negative rods and the most common mechanism of aminoglycoside resistance is enzymatic modification of antibiotic molecule. All ESBL positive isolates were sensitive to the amikacin (100 %) followed by imipenem (94.44 %) and nitrofurantoin (72.22 %). Antimicrobial resistance surveillance done Nepal Public Health Laboratory (NPHL) found that ESBL *E. coli* were susceptible to imipenem (98.5 %), amikacin (96.1 %) followed by nitrofurantoin (89.2 %) and chloramphenicol (90.8 %) [33]. Amikacin and nitrofurantoin can therefore be used effectively against ESBL producing isolates but these antibiotics have many limitations. High percentage of isolates were susceptible to the carbapenem. The study done by Kader and Angamuthu [34] revealed more than 89 % of the ESBL producers were susceptible to imipenem and meropenem, whereas Mekki et al. [35] found 100 % isolates sensitive to the carbapenems. The production of β-lactamase may be of chromosomal or plasmid origin [4, 36]. Plasmid mediated production is often acquired by transfer of genetic information from one organism to another. Such transferable plasmid also codes for resistant determinants to antimicrobial agents other than β-lactams [37]. Hence multidrug resistance is expected to be more common in ESBL producing organisms.

The production of ESBL pathogens like *E. coli* has an important clinical importance. It has been well recognized that poor outcome occurs when patients with serious infections due to ESBL-producing organisms are treated with antibiotics like cephalosporins and penicillins to which the organisms are resistant. The mortality rate in such patients is significantly higher than in patients treated with antibiotics to which the organism is susceptible. All patients with antibiotics failure either die or have continued sign of infections, which necessitates change in antibiotic [38]. Microbiology laboratories can play an important role in detecting and promptly reporting the isolation of ESBL-positive bacteria, since drug susceptibility data are important for the clinical management of patients infected by these organisms [39]. Clinicians, whose laboratories do not perform tests for detection of ESBLs, and report ESBL producers as resistant to cephalosporins, risk poor outcome for their patients infected with ESBL producing organisms. The detection of ESBLs in any clinical isolate has great potential significance from the point of view of infection control [38].

This study also has some limitations; the study was carried out in a hospital. The picture of the study does not necessarily reveal the picture of the whole country, therefore systematic prospective surveillance should be carried covering wide geographical region in order to obtain information on seasonal, geographical and ethnic variation of pathogens and their antibiotic susceptibility profile. Moreover, characterization of ESBL strains should be performed genotypically, so that the information can be used in fighting with increasing resistance to antimicrobials.

## Conclusion

The present study looked at the ESBL production in *E. coli* isolates in National Kidney Center in Kathmandu, Nepal*.* Using the phenotypic confirmatory test forwarded by the CLSI, relatively significant *E. coli* isolates tested were ESBL producers. Multidrug resistance to the antibiotics tested was also seen more among ESBL producer than the non-producers. In this study Enterobacteriaceae isolates showed significant resistance to the commonly prescribed drugs. In the present study 96.84 % Enterobacteriaceae were found to be multidrug resistant and 26.87 % *E. coli* were ESBL producers. These findings suggest for further study in this field including the consequences of colonization with multidrug resistant and ESBL-producing bacteria both in the community and in the hospital setting. These findings also suggest incorporating early detection mechanism of ESBL by clinical setting so that appropriate antibiotics can be used and this will also help in controlling increasing multidrug resistance due empirical therapy. With the spread of ESBL producing strains in hospitals all over the world, it is necessary to know the prevalence of ESBL positive strains in a hospital so as to formulate a policy of empirical therapy in high risk units where infections due to resistance organisms is higher.

## Abbreviations

AST: Antibiotic susceptibility testing
BA: Blood agar
CLSI: Clinical and Laboratory Standard Institute
CoNS: Coagulase-negative staphylococci
ESBL: Extended spectrum β-lactamase
ICU: Intensive care unit
MA: MacConkey agar
MHA: Mueller Hilton agar
MDR: Multidrug resistant
MSU: Mid-stream urine
NPHL: Nepal Public Health Laboratory
UTI: Urinary tract infection
